# Serum cytokine profiles in patients with myasthenia gravis

**DOI:** 10.3389/fneur.2025.1611673

**Published:** 2025-07-03

**Authors:** Xuan Wu, Huan Huan Song, Guo Rong Xu, Run Yun Li, Xiao Bin Ye

**Affiliations:** ^1^Department of Neurology, The First Affiliated Hospital of Fujian Medical University, Fuzhou, China; ^2^Department of Neurology, National Regional Medical Center, Binhai Campus of the First Affiliated Hospital, Fujian Medical University, Fuzhou, China; ^3^Department of Neurology and Institute of Neurology of First Affiliated Hospital, Institute of Neuroscience, Fujian Medical University, Fuzhou, China

**Keywords:** anti-AChR antibodies, cytokines profiles, thymoma, myasthenia gravis, biomarkers

## Abstract

**Background:**

Cytokines play a crucial role in instigating inflammation and generating pathogenic autoantibodies at the neuromuscular junction in individuals suffering from myasthenia gravis (MG). The objective of this study is to investigate the cytokine profiles among patients grappling with MG.

**Methods:**

This study recruited patients with unstable MG and healthy controls from the First Affiliated Hospital of Fujian Medical University during the period spanning January 2021 to December 2022. We evaluated IL-1β, IL-2, IL-4, IL-5, IL-6, IL-8, IL-10, IL-12P70, IL-17, IFN-γ, IFN-α, and TNF-α in the serum using the Flow Cytometric Bead Array (CBA) technique.

**Results:**

A total of 104 patients and 54 healthy controls were included in the study. Notably, serum levels of interleukin (IL)-1β, IL-2, IL-10, and IL-17 exhibited significant elevation in unstable MG patients when compared to the healthy control group. Furthermore, levels of IL-1β and IL-5 were notably higher in unstable MG patients who tested negative for acetylcholine receptor (AChR) antibodies when compared to their AChR-antibody positive counterparts (*P* < 0.05). In AChR-antibody positive patients, there was a statistically significant decrease in IL-1β, IL-2, IL-4, IL-6, IL-8, IL-10, IL12-P70, IFN-γ, and IFN-α upon improvement. There was no discernible variation in MG patients at an unstable stage regardless of their onset time. Additionally, there was no statistically significant differences between pre- and post-thymectomy in thymoma-associated MG (TAMG).

**Conclusion:**

Individuals with unstable MG appear to demonstrate elevated levels of serum IL-1β, IL-2, IL-10, and IL-17 compared to healthy individuals. Furthermore, among MG subgroups, those testing negative for antibodies, tend to display increased levels of IL-1β and IL-5. These serum cytokine profiles may hold promise as potential biomarkers for stratifying MG patients in clinical settings.

## Introduction

Myasthenia gravis (MG) represents the most prevalent neuromuscular junction disorder, primarily instigated by pathogenic autoantibodies targeting components of the postsynaptic muscle endplate. Clinically, MG presents as muscle weakness and fatigability and is frequently accompanied by thymic abnormalities, such as follicular hyperplasia or thymoma (1, 2). In China, MG exhibits an incidence rate of 0.68 per 100,000 person-years (3). It is noteworthy that over 80% of MG patients harbor autoantibodies directed against the acetylcholine receptor (AChR) (4). These autoantibodies accelerate endocytosis, leading to AChR degradation and complement-mediated destruction of the neuromuscular junction, consequently resulting in a reduction of acetylcholine and sodium channel receptors (5). MG is categorized into two main forms: ocular myasthenia gravis (OMG) and generalized myasthenia gravis (GMG), based on the muscles affected.

Cytokines play a crucial role as critical mediators that intricately govern immune and inflammatory responses through complex networks. Additionally, they function as biomarkers for a variety of diseases (6). Additionally, T cells, B cells, plasma cells, as well as cytokines, play crucial roles in the production of AChR antibodies in MG, representing upstream components in the immunological pathogenesis of MG (3, 7, 8). Several cytokines, including interleukin (IL)-17, CXCR5, IL-21, and IL-6, among others, have been identified in the production of pathogenic autoantibodies and the inflammation observed in MG (9–11). However, numerous cytokines, characterized by their exceedingly low concentrations, remain largely unexplored. Moreover, there is limited research concerning the relationship between cytokines and distinct disease subgroups.

Thus, the primary objective of this study was to ascertain the serum levels of cytokines in patients afflicted with unstable MG, with further subdivision based on the distribution of weakness, AChR antibodies, and the presence of thymoma. Simultaneously, the alterations in cytokine levels were monitored in response to disease remission and following thymoma surgery. The study aimed to unveil the serum cytokine profiles characterizing unstable MG and potentially establish novel biomarkers for the screening and stratification of MG patient's in future clinical practice.

## Methods

### Study design and participants

This study recruited patients with unstable MG at the First Affiliated Hospital of Fujian Medical University between January 2021 and December 2022. The diagnosis of MG relied on clinical manifestations of fluctuating and fatigable weakness in voluntary muscles, in conjunction with at least one of the following criteria: (1) positive serum anti-AChR; (2) a decrease of more than 10% in compound muscle action potentials during repetitive nerve stimulation at frequencies of 3–5 Hz; (3) a definitive positive response to neostigmine. All patients had already been tested with AChR, muscle-specific tyrosine kinase protein (MuSK) and low-density lipoprotein receptor-related protein 4 (LRP4) antibodies. New-onset MG was defined as < 12 months since disease onset. Unstable MG was defined as either (1) the new-onset MG symptoms with no improvement since initial disease onset, or (2) suffered worse or exacerbation of post-intervention status (PIS) within 1 month (12). Exclusion criteria encompassed the presence of (1) positive serum MuSK antibodies or LRP4 antibodies; (2) concurrent uncontrolled autoimmune disorders, such as hyperthyroidism, Systemic Lupus Erythematosus (SLE), and Sjogren's syndrome; and (3) acute infections. A group of individuals without autoimmune diseases, infections, or severe underlying conditions were recruited from the health center as healthy controls.

All patients were subsequently followed up, with blood samples being collected again post-treatment upon reaching minimal manifestations status or better. Patients with thymoma underwent additional sampling following thymoma surgery.

This study garnered approval from the Ethical Review Board for Medical Research and Clinical Technology Application, Ethics Committee of the First Affiliated Hospital of Fujian Medical University. All participants provided written informed consent.

### Procedures

Patients were recruited from the myasthenia gravis registration cohort at our hospital, and healthy controls were matched based on gender and age criteria corresponding to the enrolled cases. Blood samples were collected from both patients and healthy participants.

Fresh Ethylene Diamine Tetraacetic Acid (EDTA) anticoagulated whole blood samples were obtained, and serum was subsequently separated. The levels of IL-1β, IL-2, IL-4, IL-5, IL-6, IL-8, IL-10, IL-12P70, IL-17, IFN-γ, IFN-α, and TNF-α were assessed using the Flow Cytometric Bead Array (CBA) technique (13). All procedures strictly adhered to the manufacturer's instructions. If the measurement falls below the minimum detection limit, it should be recorded as the minimum detectable value. Data, including gender, age, age of onset, disease duration, MGFA Postintervention Status, anti-AChR antibody titer, anti-MuSK antibody titer, anti-LRP4 antibody titer, and the presence of thymus at the time of serum sampling, were collected.

### Statistical analysis

Statistical analysis was performed using SPSS version 26.0 (IBM Corp., USA) for data analysis, and Prism 9 (GraphPad, USA) was used for generating graphs. Continuous data that adhered to a normal distribution were presented as means ± standard deviation (SD) and compared using independent *t*-tests and paired-samples *t*-tests. Continuous data exhibiting a skewed distribution were expressed as median (range) and compared using the Mann-Whitney test and Wilcoxon test. Categorical data were presented as *n* (%) and compared using the Fisher exact test. Subgroup analyses were conducted between OMG and GMG, AChR-antibody positive and AChR-antibody negative, and thymoma-associated MG (TAMG) and patients without thymoma (Non-thymomatous MG). AChR-antibodies were assessed using the radio immunoprecipitation assay (RIA). All TAMG cases were confirmed via surgical procedures and pathology, whereas patients without thymoma exhibited no signs of thymoma on chest CT. A two-sided *P-value* of < 0.05 was deemed statistically significant.

## Results

A total of 104 patients were enrolled. Out of the 104 patients, 46 (44.2%) were male, and 58 (55.8%) were female. The age at disease onset ranged from 11 to 80 years, with a median of 49 years. The mean age at enrollment was 48.5 ± 16.2 years. The duration of the disease ranged from 1 to 252 months, with a median of 4 months. All patients were in an unstable state when enrolled. Among these patients, 64 (61.5%) were new-onset with MG, 15 (14.5%) experienced worse, and 25 (24.0%) were in an exacerbation phase. A total of 88 patients had not used glucocorticoids or immunosuppressant's for at least 6 consecutive months before enrollment, while nine patients had used glucocorticoids only, three patients had used tacrolimus, and four patients had received combined therapy with glucocorticoids and immunosuppressant's (three with tacrolimus and one with azathioprine). There were 25 cases of thymoma-associated MG (TAMG), with 17 cases had not yet undergone thymus surgery at the time of enrollment, and eight had previously undergone thymectomy at the time of enrollment. Among the 17 patients who had not yet undergone thymus surgery at the time of enrollment, 10 patients subsequently underwent serum cytokine detection after the thymus surgery again. The demographic and clinical characteristics of the patients are presented in Tables 1, 2.

**Table 1:** Patient characteristics.

**Characteristics**	**MG group (*n* = 104)**	**Healthy group (*n* = 54)**	***p-value***	**Subgroup 1**			**Subgroup 2**			**Subgroup 3**		
				**OMG (*****n*** = **41)**	**GMG (*****n*** = **63)**	***p-value***	**AChR (**+**) (*****n*** = **84)**	**AChR (–) (*****n*** = **20)**	***p-value***	**TAMG (*****n*** = **25)**	**non-thymomatous MG (*****n*** = **79)**	***p-value***
Age of onset (years)	49 (11–80)	–	–	51 (17–77)	46 (11–80)	0.151	51 (11–80)	34.5 (17–57)	0.002	54 (23–71)	45 (11–80)	0.258
Age (years)	48.5 ± 16.2	47.41 ± 12.1	0.645	50.2 ± 16.2	47.3 ± 16.2	0.367	50.9 ± 16.4	38.4 ± 10.8	< 0.001	51.6 ± 12.7	47.5 ± 17.0	0.202
Disease duration (months)	4 (1–252)	–	–	2 (1–96)	8 (1–252)	0.009	4 (1–252)	5 (1–216)	0.801	5 (1–252)	4 (1–240)	0.835
Gender			0.506			0.451			0.672			0.174
Male	46(44.2%)	27 (50%)		20 (48.8%)	26 (41.3%)		38 (45.2%)	8 (40.0%)		14 (56.0%)	32 (40.5%)	
Female	58(55.8%)	27 (50%)		21 (51.2%)	37 (58.7%)		46 (54.8%)	12 (60.0%)		11 (44.0%)	47 (59.5%)	
Distribution of weakness						–			< 0.001			0.001
OMG	41 (39.4%)	–	–	–	–		25 (29.8%)	16 (80.2%)		2 (8.0%)	39 (49.4%)	
GMG	63 (60.6%)	–	–	–	–		59 (70.2%)	4 (20.0%)		23 (92.0%)	40 (50.6%)	
AChR-antibody						< 0.001			–			0.005
Positive	84 (80.8%)	–	–	25 (61.0%)	59 (93.7%)		–	–		25 (100%)	59 (74.7%)	
Negative	20 (19.2%)	–	–	16 (39.0%)	4 (6.3%)		–	–		0 (0)	20 (25.3%)	
Thymoma						0.001			0.022			–
TAMG	25 (24.0%)	–	–	2 (4.9%)	23 (36.5%)		25 (29.8%)	0 (0)		–	–	
Without thymoma	79 (76.0%)	–	–	39 (95.1%)	40 (63.5%)		59 (70.2%)	20 (100%)		–	–	

**Table 2:** Medication situation of unstable MG patients.

**Status of the disease**	**Glucocorticoids**	**Tacrolimus**	**Glucocorticoids and tacrolimus**	**Glucocorticoids and azathioprine**	**No glucocorticoids or immunosuppressant**	**Total**
new-onset	2	0	1	0	61	64 (61.5%)
worse	2	1	1	0	11	15 (14.5%)
exacerbation	5	2	1	1	16	25 (24.0%)
total	9	3	3	1	88	104

**Figure 1:**
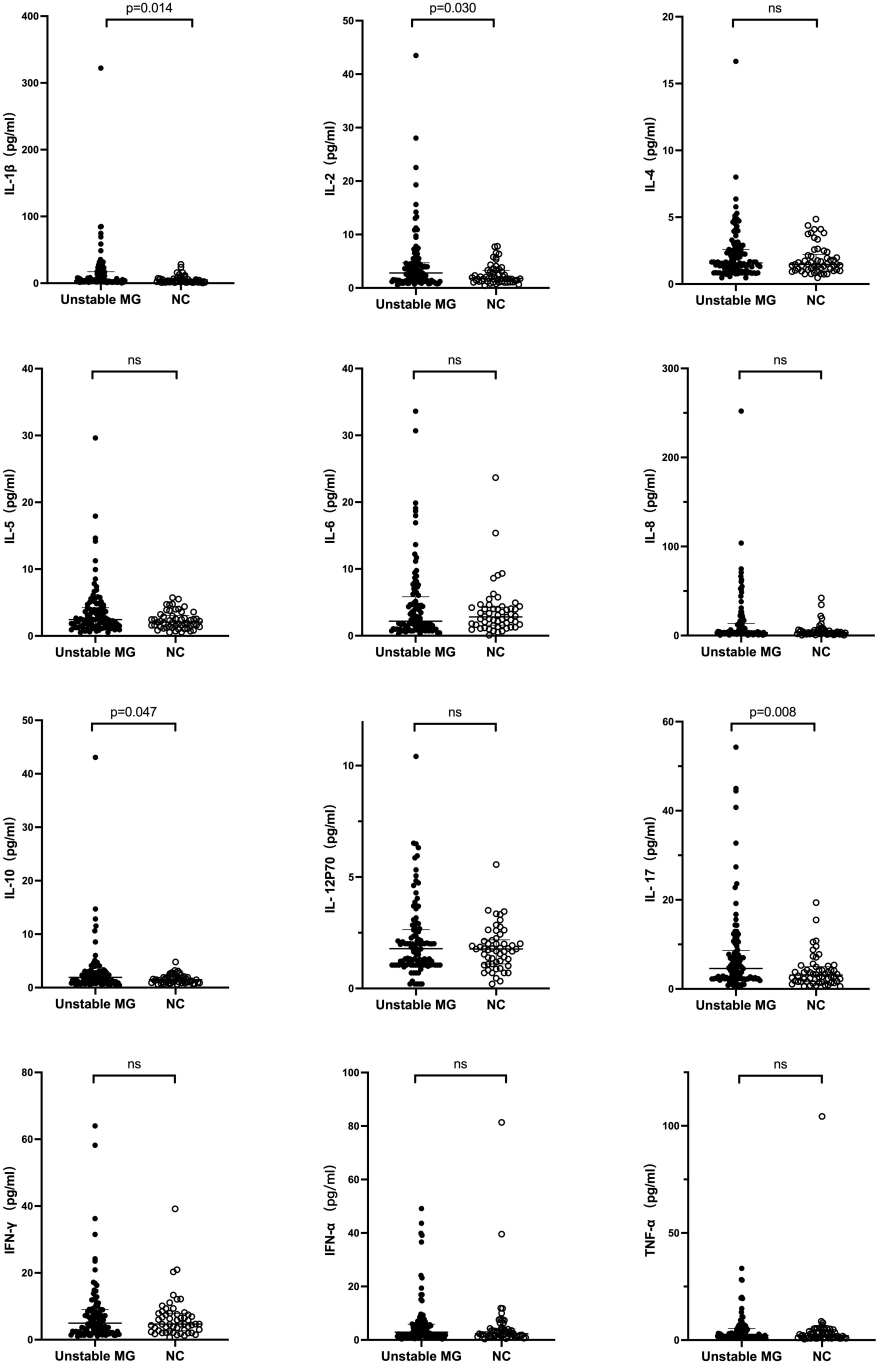
Serum cytokine levels in unstable myasthenia gravis (MG) patients and healthy controls.

Among the 12 different serum cytokines, significant differences were observed between all patients with unstable MG and healthy controls in the levels of IL-1β, IL-2, IL-10, and IL-17 (*P* < 0.05). Patients with unstable MG exhibited higher levels of these cytokines compared to healthy controls (Table 3; Figure 1). While IL-5 and IL-8 levels showed a tendency to be higher (*P* < 0.10) in patients with unstable MG than in healthy controls, these differences did not reach statistical significance. There were no statistically significant differences observed in any of the 12 cytokines between new-onset MG patients and patients experiencing worse or exacerbation.

**Table 3:** Serum cytokine profiles in patients with unstable MG and healthy controls.

**Cytokines, pg/ml**	**MG group, *n* = 104**	**Healthy group, *n* = 54**	***p-value***	**OMG, *n* = 41**	**GMG, *n* = 63**	***p-value***	**AChR (+), *n* = 84**	**AChR (–), *n* = 20**	***p-value***	**TAMG, *n* = 25**	**non-thymomatous MG, *n* = 79**	***p-value***
IL-1β	3.97 (0.41–322.17)	2.74 (0.39–28.19)	0.014	5.51 (0.41–322.17)	3.73 (0.95–74.54)	0.423	3.59 (0.59–322.17)	10.77 (0.47–84.89)	0.029	3.59 (0.95–322.17)	4.68 (0.4–84.89)	0.825
IL-2	2.80 (0.59–43.50)	1.78 (0.42–7.81)	0.030	2.97 (0.59–43.5)	2.59 (0.74–22.53)	0.653	2.62 (0.59–43.5)	3.62 (0.79–28.03)	0.13	2.41 (0.77–43.5)	2.97 (0.59–28.03)	0.643
IL-4	1.58 (0.47–16.66)	1.47 (0.47–4.86)	0.606	1.59 (0.47–5.1)	1.55 (0.47–16.66)	0.842	1.56 (0.47–16.66)	1.62 (0.56–5.10)	0.827	1.86 (0.47–8.01)	1.52 (0.47–16.66)	0.337
IL-5	2.45 (0.52–29.62)	2.08 (0.54–5.74)	0.062	2.96 (0.52–17.92)	2.15 (0.62–29.62)	0.13	2.19 (0.52–29.62)	3.57 (0.54–17.92)	0.010	1.98 (0.62–14.64)	2.66 (0.52–29.62)	0.123
IL-6	2.18 (0.28–33.61)	2.83 (0.07–23.66)	0.703	2.60 (0.44–30.68)	1.86 (0.28–33.61)	0.569	1.85 (0.28–19.87)	3.04 (0.44–33.61)	0.175	2.93 (0.72–12.22)	2.11 (0.28–33.61)	0.413
IL-8	3.36 (0.44–252.08)	3.22 (0.44–42.27)	0.063	3.36 (0.44–252.08)	3.36 (0.44–103.84)	0.375	3.36 (0.44–252.08)	3.81 (0.44–70.87)	0.325	3.36 (0.74–74.9)	3.36 (0.44–252.08)	0.644
IL-10	1.92 (0.45–43.06)	1.49 (0.62–4.79)	0.047	1.89 (0.50–43.06)	1.94 (0.45–11.52)	0.892	1.60 (0.45–43.06)	2.43 (0.68–14.7)	0.137	1.98 (0.45–43.06)	1.90 (0.45–14.7)	0.755
IL-12P70	1.79 (0.20–10.41)	1.77 (0.20–5.56)	0.514	2.01 (0.20–10.41)	1.33 (0.20–6.5)	0.092	1.79 (0.20–10.41)	1.86 (0.20–4.74)	0.814	2.04 (0.20–6.49)	1.72 (0.20–10.41)	0.909
IL-17	4.59 (0.58–54.26)	3.10 (0.58–19.38)	0.008	6.04 (0.58–45.00)	3.82 (0.58–54.26)	0.202	3.80 (0.58–54.26)	7.37 (0.58–32.74)	0.088	2.81 (0.58–40.75)	4.94 (0.58–54.26)	0.056
IFN-γ	4.97 (1.04–64.01)	4.68 (0.70–39.17)	0.856	4.07 (1.04–31.5)	5.60 (1.34–64.01)	0.277	5.40 (1.04–64.01)	3.96 (1.10–58.2)	0.233	4.68 (1.34–64.01)	4.97 (1.04–58.2)	0.939
IFN-α	2.92 (0.36–49.15)	2.32 (0.36–81.37)	0.114	2.82 (0.36–49.15)	3.02 (0.47–43.61)	0.855	3.09 (0.36–49.15)	2.65 (0.74–16.93)	0.792	4.58 (0.89–23.22)	2.80 (0.36–49.15)	0.498
TNF-α	2.14 (0.82–33.45)	2.03 (0.47–104.37)	0.155	2.40 (0.82–14.74)	1.93 (0.82–33.45)	0.774	1.94 (0.82–33.45)	2.55 (0.82–19.97)	0.846	2.68 (0.82–19.62)	2.01 (0.82–33.45)	0.542

Furthermore, the levels of IL-1β and IL-5 were significantly higher in the subgroup of MG patients with AChR-antibody negative compared to those with AChR-antibody positive (*P* < 0.05). However, there was no significant difference in any of the 12 cytokines between subgroups of OMG and GMG, TAMG, and non-thymomatous MG (all *P* > 0.05) (Table 3; Figure 1).

**Figure 2:**
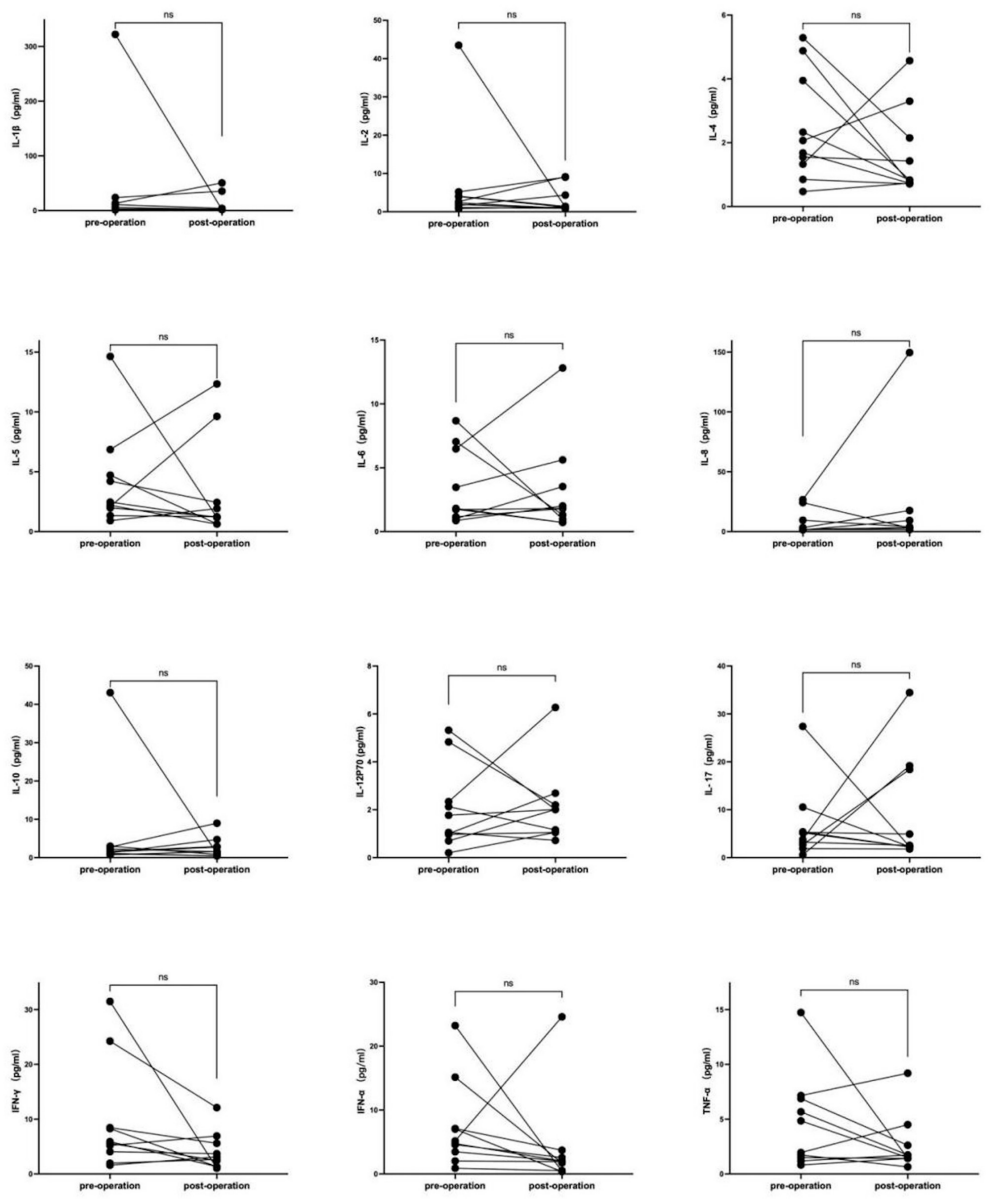
Serum cytokine change curves in MG patient's pre- and post- thymus surgery. Cytokines detection was performed in 10 patients before and after thymectomy, there was no significant difference in any of the 12 cytokines before and after thymectomy.

Cytokines detection was performed in 10 patients before and after thymectomy, the characteristics of these patients are presented in Table 4. There were no significant differences in any of the 12 cytokines before and after thymectomy, the change curves are shown in Figure 2.

**Table 4:** Demographics of the patients detected both before and after thymectomy.

**Patient**	**Gender**	**Age**	**Status of the disease pre-operation**	**Status of the disease post-operation**	**Time between two detections (months)**	**Time between the second test and thymectomy (months)**	**Thymus pathology**
1	M	46	worse	Unchanged	11	7	Microthymoma
2	F	26	new-onset	Minimal manifestations	7	5	B3
3	M	23	new-onset	Improved	1	1	B2
4	F	42	new-onset	Pharmacologic remission	11	10	B3
5	F	54	new-onset	Improved	11	10	B3
6	M	36	new-onset	Improved	8	4	B3
7	F	51	new-onset	Minimal manifestations	23	10	B3
8	M	63	new-onset	Improved	15	13	B1
9	F	56	new-onset	Minimal manifestations	13	8	B2
10	M	56	new-onset	Minimal manifestations	10	7	B2

**Figure 3:**
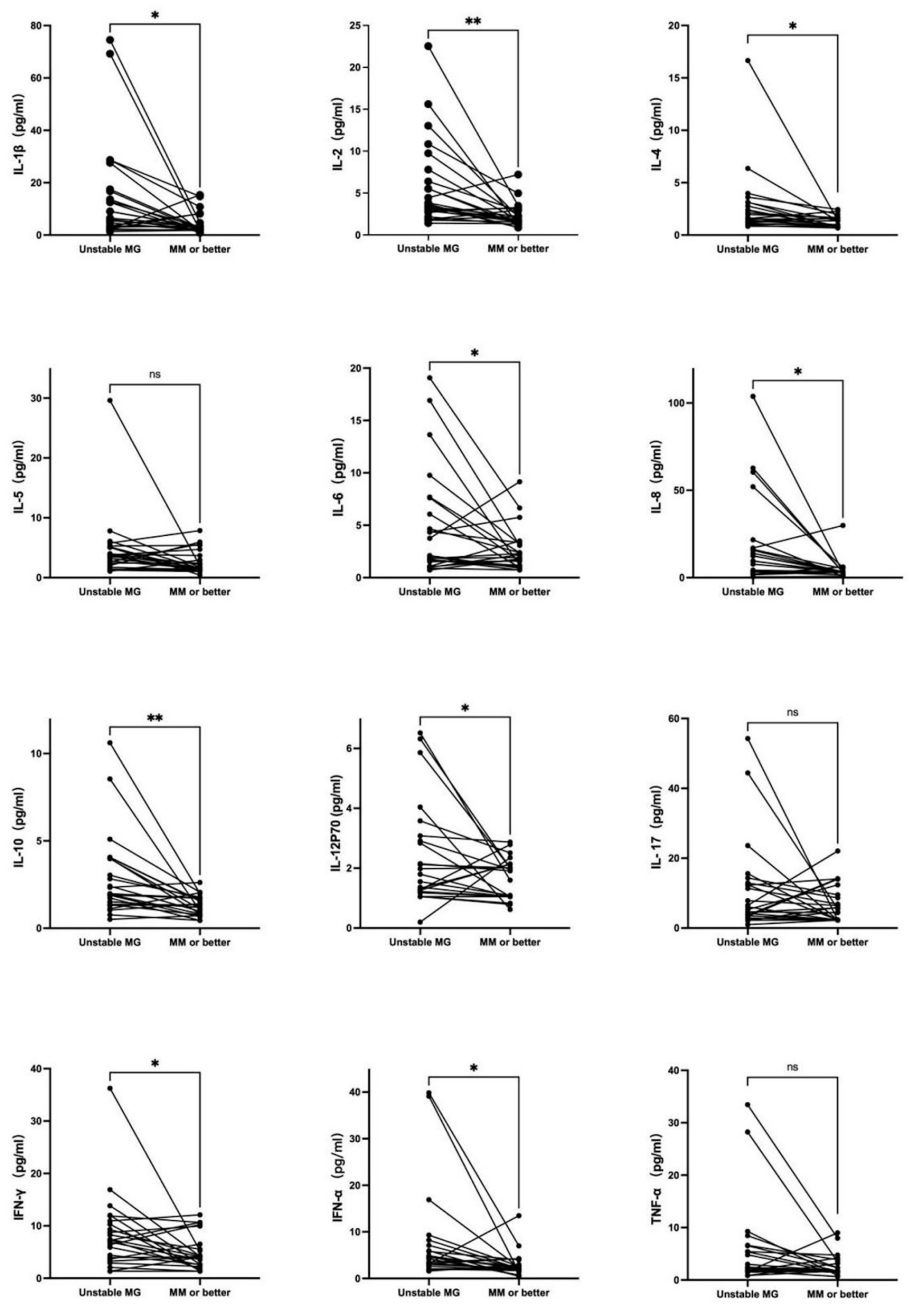
Twenty-four AChR-antibody positivite, non-thymomatous, unstable MG patients had cytokines detected again after reaching minimal manifestations status or better. IL-1β, IL-2, IL-4, IL-6, IL-8, IL-10, IL-12P70, IFN-γ, and IFN-α showed a statistically significant decrease upon reaching minimal manifestations status or better. Conversely, there was no significant change in IL-5, IL-17, and TNF-α. *P < 0.05, **P < 0.01.

Twenty-four AChR-antibody positive, non-thymomatous, unstable MG patients had cytokines detected again after reaching minimal manifestations status or better. Among these patients, two patients underwent thymectomy during the follow-up period, with pathology indicating thymic hyperplasia. IL-1β, IL-2, IL-4, IL-6, IL-8, IL-10, IL-12P70, IFN-γ, and IFN-α showed a statistically significant decrease upon reaching minimal manifestations status or better. Conversely, there was no significant change in IL-5, IL-17, and TNF-α (Figure 3).

**Figure 4:**
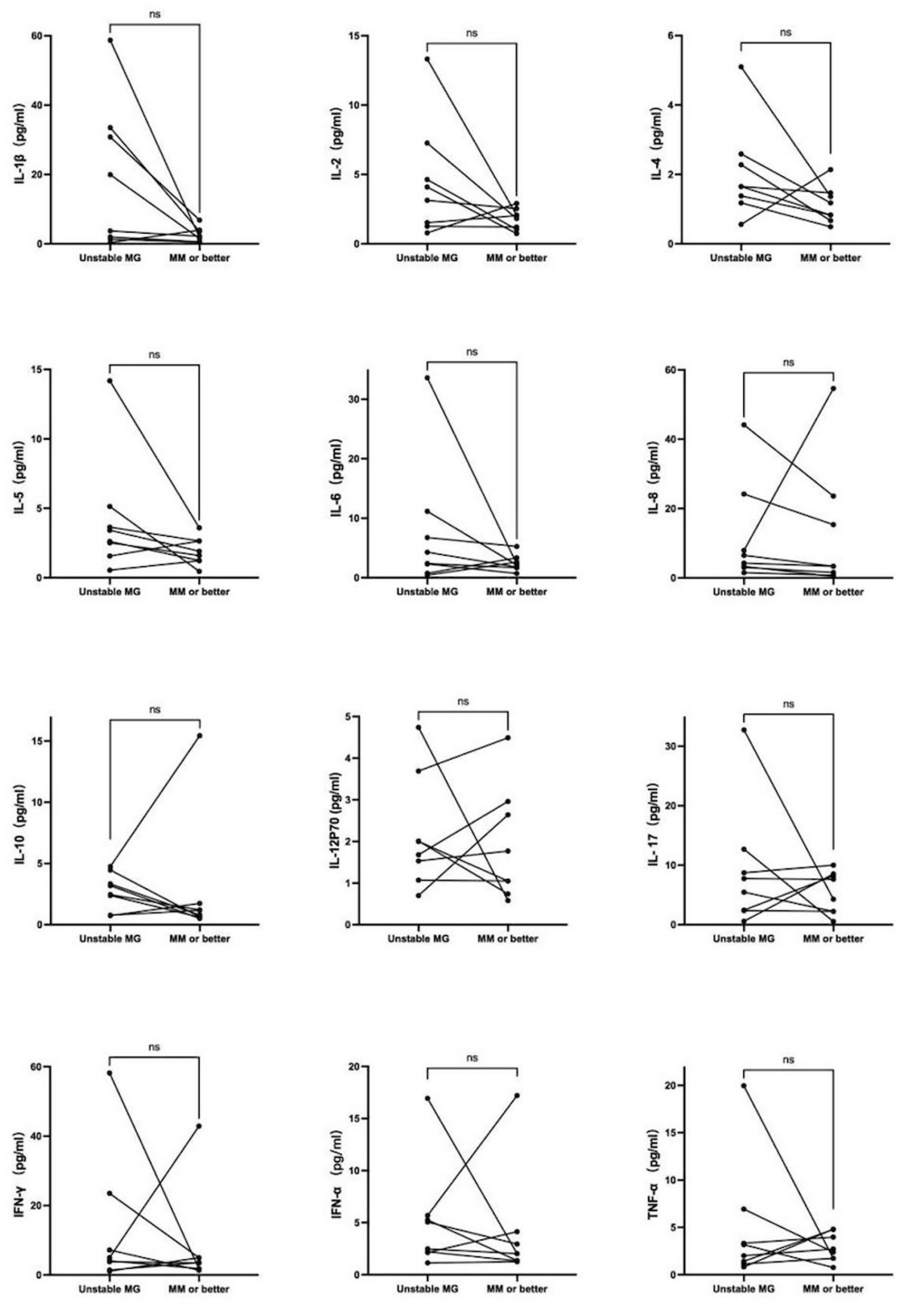
Eight AChR-antibody negative, non-thymomatous, unstable MG patients had cytokines detected again after reaching minimal manifestations status or better. No significant changes were observed in any of the 12 cytokines.

Eight AChR-antibody negative, non-thymomatous, unstable MG patients had cytokines detected again after reaching minimal manifestations status or better. No significant changes were observed in any of the 12 cytokines, as depicted in Figure 4.

## Discussion

This study observed that the serum levels of IL-1β, IL-2, IL-10, and IL-17 were potentially elevated in patients with unstable MG when compared to healthy individuals. Additionally, the serum levels of IL-1β and IL-5 exhibited significant increases in the subtype of MG patients with AChR-antibody negative as opposed to those with AChR-antibody positive. Additionally, a statistically significant decrease was observed in the serum levels of IL-1β, IL-2, IL-4, IL-6, IL-8, IL-10, IL-12P70, IFN-γ, and IFN-α upon reaching minimal manifestations status or better. These findings provide support for the existence of a serum cytokine profile that could serve as potential biomarkers for stratifying MG patients in clinical practice.

IL-1β plays a pivotal role as a proinflammatory cytokine involved in the regulation of the innate immune response. It has been implicated in perpetuating immune responses and contributing to conditions such as diabetic retinopathy, rheumatoid arthritis, multiple sclerosis, and Crohn's disease (14–16). Huang et al. revealed a critical role for IL-1β in inducing MG in mice, supporting its involvement in the pathogenesis of MG in humans (11). This study also demonstrated a significant increase in IL-1β levels among MG patients in an unstable stage and a statistically significant decrease when reaching stable, consistent with previous research. IL-2 serves as a vital T cell growth factor, essential for T cell proliferation and the generation of effector and memory cells (18–20). Some drugs, such as tacrolimus and cyclosporine, are used in MG primarily by inhibiting IL-2 signaling transcription (10, 21). Xiao's research demonstrated increased serum IL-2 levels in AChR-positive GMG (6), aligning with our study's findings. The role of IL-10 in MG is somewhat controversial, as it possesses both inflammatory and immunosuppressive properties (17, 22). In the present study, it was observed that IL-10 levels were markedly elevated in individuals with unstable MG compared to healthy controls, and a statistically significant reduction was noted upon achieving stability. IL-17 functions as the primary effector of Th17 cells and has been reported to be up-regulated and associated with clinical parameters of MG (22, 23), in line with the findings of this study, where IL-17 levels were significantly higher in patients with unstable MG compared to healthy controls.

IL-4 and IL-5 are categorized as type 2 cytokines (24), and although they have received relatively less attention in MG research, several studies have indicated elevated IL-4 and IL-5 levels in MG patients (9, 25–28). However, one study suggested that IL-4 may have a protective role in electromyography (EMG) (29). In our study, we did not observe significant differences in serum levels of IL-4 and IL-5 between MG patients and healthy controls. Nonetheless, there was a tendency for IL-5 levels to be higher in MG patients, and a statistically significant decrease in IL-4 levels was observed upon reaching stability. IL-8 is a type 1 cytokine and has been reported to be elevated in MG patients in multiple studies (26, 30). IL-12, an essential type 1 immune activation cytokine, consists of both the biologically inactive form (IL-12p40) and the active form (IL-12p70). The role of these two cytokines in MG has not been thoroughly investigated. While our study did not identify significant differences in serum levels of IL-8 and IL-12 between unstable MG patients and healthy controls, there was a trend toward higher IL-8 levels in MG patients, and a statistically significant decrease in IL-8 and IL-12p70 levels was observed upon reaching stability. IL-6 is a prototypical cytokine known for its pleiotropic effects on inflammation, immune response, and hematopoiesis (31). It can contribute to sustaining chronic inflammation and B cell maturation (32, 33). Studies by Revital Aricha and Akiyuki Uzawa indicated that blocking IL-6 could suppress experimental autoimmune myasthenia gravis (EAMG) and that high serum IL-6 levels were associated with disease activity in MG, respectively (34, 35). Tocilizumab, an anti-IL-6 receptor humanized monoclonal antibody, has shown effectiveness in MG cases refractory to rituximab and a prospective, open-label, single-arm study (36, 37). However, our study did not reveal significantly higher serum levels of IL-6 in unstable MG patients compared to healthy controls, but a statistically significant decrease in IL-6 levels was observed upon reaching stability. IFN-γ, IFN-α, and TNF-α are key cytokines produced by innate immune cells (17, 22). Our study found no significant differences in the levels of these three cytokines between unstable MG patients and healthy controls, but a statistically significant decrease in IFN-γ and IFN-α level was observed upon reaching stability. MG is commonly associated with autoantibodies targeting AChR, MuSK, and LRP4 (38–40).

Antibody-negative myasthenia gravis refers to cases of MG in which AChR, MuSK, or LRP4 antibodies are undetectable. Additional antibodies, such as anti-agrin and cortactin antibodies, are sometimes present in conjunction with other autoantibodies (41, 42). However, the functional relationship of these additional antibodies to other targeted proteins remains unclear. In our study, we observed that unstable MG patients who tested negative for AChR antibodies had higher serum levels of IL-1β and IL-5 compared to those who tested positive for AChR antibodies. Several cytokines exhibited a decrease in patients with minimal manifestations or improved state in AChR antibody-positive MG, whereas no such trend was observed in antibody-negative MG, suggesting distinct pathogenesis in the latter.

There were no statistically significant differences observed in any of the 12 cytokines between new-onset MG patients and patients experiencing worse or exacerbation, indicating that there was no discernible variance in the serum cytokines levels of patients with MG in an unstable stage, regardless of their onset time.

In the study, no statistically significant differences were observed in the levels of any of the 12 cytokines before and after thymectomy in patients with TAMG, suggesting that thymus may not have a substantial impact on serum cytokine levels.

This study has several limitations. Firstly, it's important to consider that AChR antibodies are the pathogenic antibodies in MG, and cytokines may play a role in pathogenesis by influencing the number of AChR-specific antibody-secreting cells. Therefore, our study primarily focused on the concentration of certain cytokines in unstable MG patients but did not delve into their effects on antigen-specific B cells, T cells, and plasma cells. Additionally, elevated levels could reflect downstream inflammation rather than disease-driving factors. Secondly, our data revealed that serum cytokine levels did not adhere to a normal distribution, displaying significant differences among different patients, with a small portion of patients registering levels below the detectable range. Thirdly, the cytokine network is intricate, with cytokines mutually influencing each other. We believe that only factors directly impacting the secretion of AChR antibodies hold the potential to predict the disease's course. Notably, Robert et al. developed an *in vitro* model that could serve as a paradigm for studying antibody-mediated conditions like neuromyelitis optica spectrum disorder (NMOSD) (43). This model offers the opportunity to apply condition-specific approaches to patients with antibody-mediated diseases, predicting conditions conducive to producing antibody-secreting cells (ASCs) and specific antibodies. In their work, Robert et al. observed that the percentage of ASCs was higher in conditions involving IL-1β and TNF-α compared to other conditions, and within these conditions, the proportion of detectable AQP4-IgGs was also higher, suggesting that IL-1β and TNF-α may enhance the production of serum AQP4 antibodies. This model has also been employed in the context of N-methyl-D-aspartate receptor (NMDAR) antibody encephalitis (44). In our study, we identified significant differences in serum levels of IL-1β, IL-2, IL-10, and IL-17 between unstable MG patients and healthy controls. However, in-depth *in vitro* models will be necessary for further validation in the future. Finally, our study is designed as a cross-sectional analysis, and not all patients underwent repeated detection during the follow-up period.

## Conclusions

In summary, it appears that unstable MG patients exhibit elevated serum levels of IL-1β, IL-2, IL-10, and IL-17 compared to healthy individuals. Furthermore, the subtype of MG characterized by AChR-antibody negativity tends to demonstrate higher levels of IL-1β and IL-5 in comparison to those with AChR-antibody positivity. IL-1β, IL-2, IL-4, IL-6, IL-8, IL-10, IL-12P70, IFN-γ, and IFN-α showed a statistically significant decrease upon reaching minimal manifestations status or better in AChR-antibody positive, non-thymomatous, unstable MG patients. These findings shed light on the cytokine profiles present in the serum of unstable MG patients and suggest the potential utility of these cytokines as novel biomarkers for the screening and stratification of MG patient's in future clinical practice.

## Data Availability

The raw data supporting the conclusions of this article will be made available by the authors, without undue reservation.
